# Release characteristics of overpressurised gas from complex vents: implications for volcanic hazards

**DOI:** 10.1007/s00445-020-01407-2

**Published:** 2020-10-02

**Authors:** Markus Schmid, Ulrich Kueppers, Valeria Cigala, Jörn Sesterhenn, Donald B. Dingwell

**Affiliations:** 1 Ludwig-Maximilians-Universität (LMU) München, 80333 Munich, Germany; 2 Universität Bayreuth, 95447 Bayreuth, Germany

**Keywords:** Explosive eruptions, Crater asymmetry, Vent asymmetry, Gas jets, Inclined jets, Tilted eruptions

## Abstract

Many explosive volcanic eruptions produce underexpanded starting gas-particle jets. The dynamics of the accompanying pyroclast ejection can be affected by several parameters, including magma texture, gas overpressure, erupted volume and geometry. With respect to the latter, volcanic craters and vents are often highly asymmetrical. Here, we experimentally evaluate the effect of vent asymmetry on gas expansion behaviour and gas jet dynamics directly above the vent. The vent geometries chosen for this study are based on field observations. The novel element of the vent geometry investigated herein is an inclined exit plane (5, 15, 30° slant angle) in combination with cylindrical and diverging inner geometries. In a vertical setup, these modifications yield both laterally variable spreading angles as well as a diversion of the jets, where inner geometry (cylindrical/diverging) controls the direction of the inclination. Both the spreading angle and the inclination of the jet are highly sensitive to reservoir (conduit) pressure and slant angle. Increasing starting reservoir pressure and slant angle yield (1) a maximum spreading angle (up to 62°) and (2) a maximum jet inclination for cylindrical vents (up to 13°). Our experiments thus constrain geometric contributions to the mechanisms controlling eruption jet dynamics with implications for the generation of asymmetrical distributions of proximal hazards around volcanic vents.

## Introduction

Explosive volcanic eruptions are among the most energetic displays of Earth’s internal forces. They pose continual threats to life and infrastructure. Such eruptions are fuelled by gas overpressure, which derives from volatile oversaturation of magma and its resultant degassing, sometimes combined with external volatiles such as vaporised meteoric water (Mayer et al. 2015). The overpressure driving melt vesiculation can be released explosively if it exceeds the tensile strength of magma, leading to failure and fragmentation (Alidibirov and Dingwell 1996b; Dingwell and Webb 1990). As a consequence, gas-particle jets of variable gas-particle ratio and grain size distribution are emitted from vents at high velocity. Depending on jet temperature and the subsequent entrainment of ambient air, the eruption column can collapse and form pyroclastic density currents, or buoyantly rise into the atmosphere (Woods 2010).

Direct observations at volcanoes are limited to those accessible above the vent. What can be observed in the near-vent region is the result of a complex interplay of various source, path and exit conditions that affect the evolution of the related eruptive plumes. Directly above the vent, volcanic jets (composed of gas and pyroclasts) typically show the characteristics of underexpanded starting jets (e.g. Carcano et al. 2013; Kieffer and Sturtevant 1984; Woods and Bower 1995). The underlying physical principles of gas and multiphase flow have been investigated in both fluid dynamics (e.g. Arun Kumar and Rajesh 2017; Deo et al. 2007; Peña Fernández and Sesterhenn 2017; Sommerfeld 1994; Tsuji et al. 1984) and applied engineering (e.g. Gutmark et al. 1989; Hokenson 1986; Rice and Raman 1993; Yin et al. 2016). However, their applicability for volcanic systems is limited because of the flow regimes considered and/or the assumption of sustained jets.

Several field studies (e.g. Andronico et al. 2009; Gaudin et al. 2014; Taddeucci et al. 2012) have revealed that ejection processes are both dynamic and intricate, exhibiting for example significant ejection velocity variations during single eruptive pulses and complex propagation of gas-particle jets and eruption plumes. Both initially inclined eruption columns and directed explosions yielding eruption deposits on a small sector of a volcano have been observed e.g. Mount St. Helens in 1980 (Moore and Sisson 1981); Bezymianny in 1956 (Belousov et al. 2007). The 1984 eruption of Mayon Volcano, Philippines, produced a basal thrust column that had a tilt to the southeast, leading to fountain collapse and a preferential emplacement of pyroclastic flows down the south-eastern flank of the volcano (Lagmay et al. 1999). An asymmetric crater has been cited as the reason for the directed jets rather than wind or an inclined conduit driving the laterally focused propagation (Lagmay et al. 1999). Whereas atmospheric factors such as wind may exert a strong control on the buoyant phase of these plumes and add a lateral dimension to their transport, large and strongly convecting eruptive plumes may remain apparently unaffected even in the event of storms (Pinatubo 1991, Holasek et al. 1996). Similarly to Mayon, an irregular crater morphology was proposed by Cole et al. (2015) for Soufrière Hills Volcano, Lesser Antilles, as the controlling force for directed ballistics and fall out dispersal related to explosive activity on 17 September 1996, 5 December 2008 and 11 February 2010. In May 2008, the eruption of Chaiten Volcano, Chile, produced pyroclastic flows on the northern flank of the volcano that appear to have been generated by ‘directionally focused’ explosions (Major et al. 2013). Thus, it appears that directionality of eruptive products is often controlled by the geometry of craters and/or vents and is not necessarily associated with the failure of a volcanic edifice (Cole et al. 2015).

Vent geometry and its influence on eruption dynamics have been the focus of several studies so far. The vent size influences jet diameter and thereby mass eruption rate (e.g. Jessop et al. 2016; Koyaguchi et al. 2010; Ogden 2011; Saffaraval et al. 2012). Valentine (1997) suggested that narrow vents and high exit velocities favour the generation of buoyant plumes. Jessop and Jellinek (2014) investigated the effect of vent geometry on entrainment characteristics of volcanic jets. They found that air entrainment is more efficient for diverging vents because of a larger surface of the jet’s boundary layer due to particle inertia and trajectory. The effect of geometry on plume dynamics has been assessed in studies focusing on ejection velocity (e.g. Kieffer 1989; Wilson and Head 1981; Wilson et al. 1980) and jet radius (e.g. Jessop et al. 2016; Woods and Bower 1995; Woods and Caulfield 1992). A flaring vent can aid the transition from subsonic to supersonic flow (e.g. Kieffer 1989; Wilson and Head 1981; Woods and Bower 1995), assuming that the ejection velocity of gas and gas-particle mixtures is mainly governed by gas overpressure, gas mass fraction and temperature (Woods and Bower 1995). These studies showed that vent geometry can increase the surface area or the velocity of the volcanic jet and therefore enhance air entrainment favouring a buoyant plume over a collapsing column (Valentine 1997). However, they did not investigate how irregular, asymmetric vents or dynamically evolving vent geometries affect eruption dynamics.

**Fig. 1:**
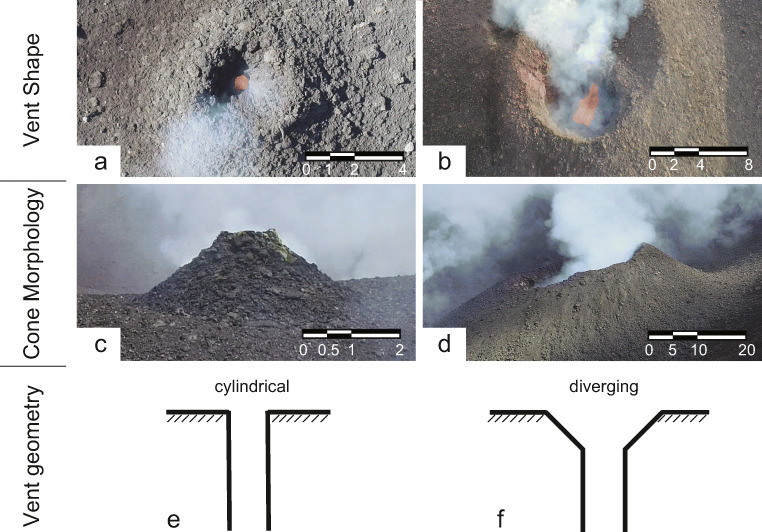
Field images acquired at Stromboli Volcano, Italy, by uncrewed aerial vehicle. The first row shows examples of the variability of vent shapes; **a** shows a circular vent, while **b** shows a highly irregular vent. The second row shows the cone morphology with symmetric (**c**) and asymmetric (**d**) vent exit heights. The third row shows sketches of the shallow subsurface with a cylindrical (**e**) and a diverging (**f**) geometry. The vent geometries used in the experiments presented in this study represent a combination of circular (**a**) vent shape with asymmetric (**d**) vent exit heights with either a cylindrical (**e**) or diverging (**f**) subsurface geometry

Volcanic edifices have been observed with highly variable topography, notched craters and slopes, hosting one or more open or (partially) clogged vents of variable shape (Fig. 1). The geometries are subject to rapid changes during a single eruption or throughout the course of several events. To date, irregular vent geometry, varying fragmentation depth and/or directionality of the underlying explosion source have been inferred as causes of asymmetric dispersal of material in scaled experiments (e.g. Graettinger et al. 2015; Graettinger et al. 2014; Valentine et al. 2012). So far, only a small number of studies have investigated the effects of vent enlargement on the dynamics of jets (Solovitz et al. 2014) and only for sustained jets. Jessop et al. (2016) tested the probability of column collapse based on the shape of the vent for symmetric annular and linear vents. Lagmay et al. (1999) combined observations and numerical models to link asymmetry in the crater area to preferential flow directions of pyroclastic flows. By employing computational fluid dynamics with a geometric analogue to a shock-tube setup, they simulated jet behaviour for a range of stagnation pressures. They found that the location of (partial) column collapse—and associated direction of pyroclastic density currents—is controlled by crater geometry and eruption exit pressure, but they did not consider the dynamic evolution of the exit pressure.

Volcanic explosions are sudden, instantaneous events from highly variable vents (Fig. 1) during which most if not all governing parameters such as magma textures and overpressure (i.e. gas-particle ratio), fragmentation efficiency (i.e. particle size), eruption depth and intensity (i.e. conduit and vent geometry, Fig. 1) vary and evolve with time. A holistic description of explosive eruptions has been attempted through several approaches, yet, to date, all approaches suffer from a lack of precision at some scale. Experimentally, shock-tube experiments have been used extensively to mimic such starting jets at controlled, reproducible conditions (e.g. Alidibirov and Dingwell 1996a; Anilkumar et al. 1993; Cigala et al. 2017; Dellino et al. 2014; Kieffer and Sturtevant 1984; Kueppers et al. 2006a, 2006b) and serve as a basis for numerical models (e.g. Lagmay et al. 1999; Ogden et al. 2008; Sommerfeld 1994).

Scaled shock-tube experiments have also been used to decipher the impact of source, path and exit conditions on volcanic phenomena. The poorly constrained to completely unconstrained boundary conditions of volcanic explosions (e.g. pressure, temperature, magma textures, particle concentration and grain size distribution) can be controlled in experiments and varied systematically. Cigala et al. (2017) revealed a general non-linear decay of particle exit velocity, governed by (1) tube length, (2) particle load, (3) vent geometry, (4) temperature and (5) particle size. They showed that vent geometry controls gas flow which in turn affects particle dynamics. To reveal the influence of complex geometry on gas expansion dynamics without any possible feedback from particles, pure gas jets have been the focus of this study. Accordingly, we modified two (cylinder and diverging 15°) of the radially symmetric geometries of Cigala et al. (2017) that showed the strongest influence on gas-particle ejection. The novelty is a slanted exit plane (5, 15, 30°) to decrease the level of symmetry resulting in six new vent geometries.

## Methodology

### Experimental setup

**Fig. 2:**
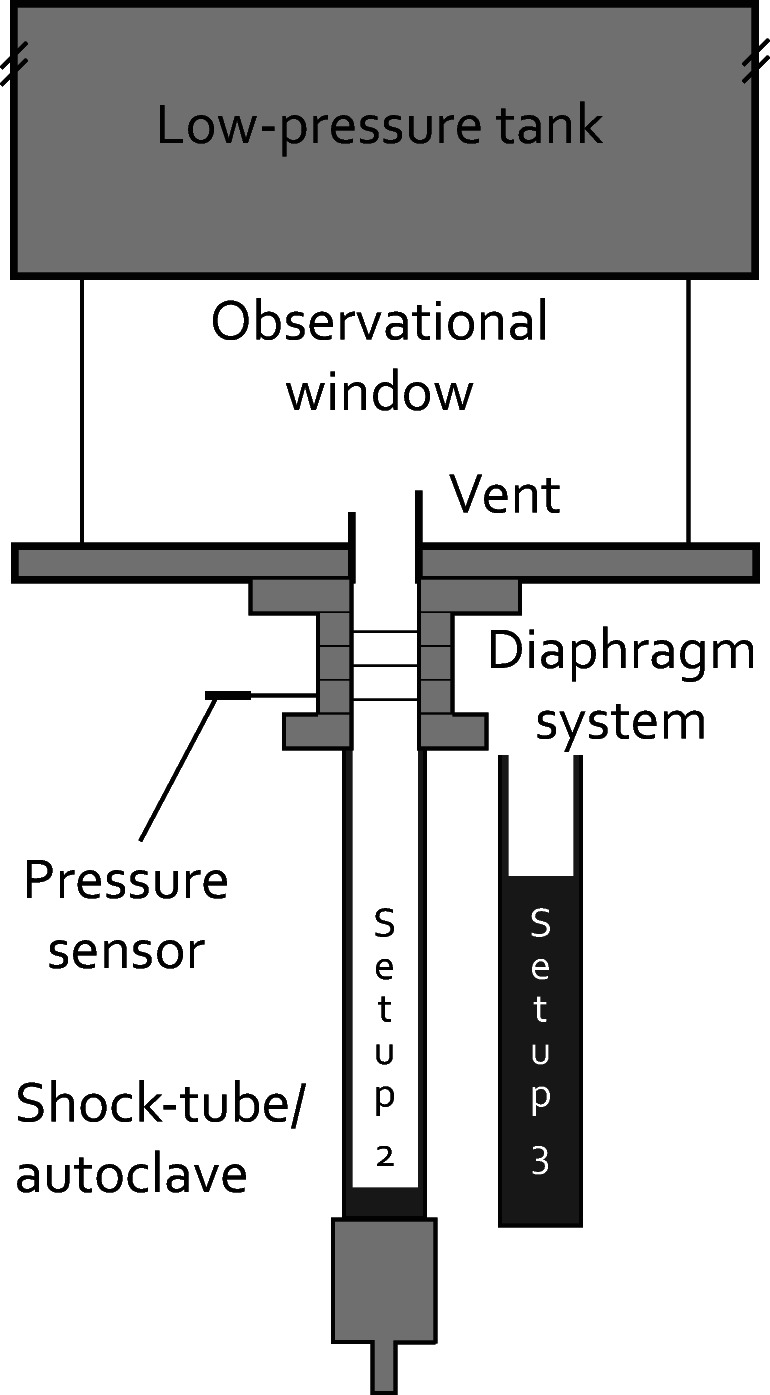
Shock-tube setup at LMU Munich consisting of the high-pressure section, the diaphragm system controlling pressurisation and the low-pressure section. The latter consist of the observational window and the tank that can be used for particle collection. Two setups with different autoclave volumes were used (setup 2: 127.4 cm^3^, setup 3: 31.9 cm^3^)

For our scaled shock-tube experiments, the ‘fragmentation bomb’ (Alidibirov and Dingwell 1996a; Kueppers et al. 2006a, 2006b; Spieler et al. 2004) has been used, with modifications building on those introduced in Cigala et al. (2017). The high-pressure high-temperature section (autoclave, Fig. 2) is separated from the low-pressure section (at ambient conditions) by a set of three diaphragms that allow for incremental pressurisation of the autoclave (with argon) to the experimental pressure.

When the desired experimental pressure in the autoclave is reached, rapid decompression of argon is triggered and the resulting pressure drop (> 1 GPa/s, Spieler et al. 2004) automatically triggers the recording system. The gas expands forming an underexpanded starting gas jet at the vent.

### This study

**Fig. 3:**
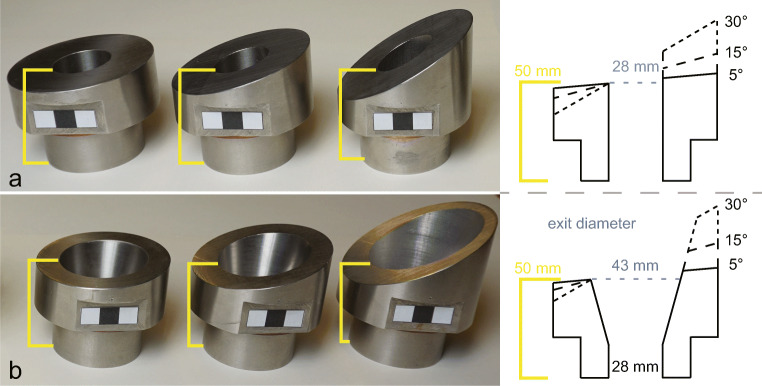
Six vent designs with bilateral symmetry. The inner geometry is either cylindrical (cyl) or a 15° diverging funnel (fun) and was selected based on the strongest impact in experiments of Cigala et al. (2017). The added vent geometry complexity is a slanted exit plane. 3a left to right: cyl05, cyl15, cyl30 and a sketch of a section through the vent. 3b left to right: fun05, fun15, fun30 and a sketch of a section through the vent. The height of the lower vent exit is identical for all geometries with 50 mm above the base of the vent (yellow mark). The upper side’s height is 52.5, 57.5 and 66 mm (cylindrical vents) and 54, 62 and 80 mm (diverging vents) above the base. Black squares used for scale 1 × 1 cm

Based on the findings of Cigala et al. (2017), we chose to adapt two geometrical configurations that showed the strongest effect on gas-particle jets: cylindrical and diverging (15°) inner geometry. For both configurations, vents with three different slant angles of the top plane (5, 15 and 30°) were fabricated (1.4305 NiCr steel, 28-mm conduit diameter, resulting in six vent geometries; see Fig 3) with bilateral symmetry. All vents were designed to reproduce conduit length as in Cigala et al. (2017), i.e. with a vent exit height of 50 mm on the lower side. The used slant angles were inspired by the geometry of eruptive vents at Stromboli Volcano, Italy (see Fig. 1). We observed circular, symmetric vents as well as irregular and asymmetric vents. One of the most frequent asymmetrical features was a varying rim height around craters and vents (see Fig. 1d). We mimic this aspect with the slanted exit plane in our vent designs. A detailed characterisation of crater and vent geometries as well as their temporal evolution over a nine-month timespan based on UAV surveys can be found in Schmid et al. (2020, in preparation).

Experiments were performed at constant temperature (25 °C), four pressure steps (5, 8, 15, 25 MPa) as well as two reservoir (autoclave) volumes (setup 2: 127.4 cm^3^, setup 3: 31.9 cm^3^; see Fig. 2). For the four starting reservoir pressures, the theoretical maximum pressure at the vent exit was calculated by applying one-dimensional isentropic theory (1.10, 1.67, 2.80 and 4.25 MPa for the cylindrical vents as well as 0.15, 0.21, 0.30 and 0.32 MPa for the diverging vents, respectively). Each experiment is triggered intentionally, generating a vertically expanding gas jet that eventually leaves the vent. Initially, the gas expands longitudinally within the shock-tube until the expansion front reaches the vent exit. From this point, the gas can expand radially forming a starting jet with turbulent eddies generated by shear between the jet and the stagnant atmosphere that will entrain ambient air. Due to decompression, the expanding argon condenses and allows visual observation of the gas dynamics. A Phantom V711 high-speed camera was used to record the experiments at 10,000 frames per second at a resolution of 1280 × 600 pixels, covering a field of view of approximately 22 × 10 cm. The videos were recorded from a point orthogonal to the symmetry plane of the vent and centred on the vent axis.

**Fig. 4:**
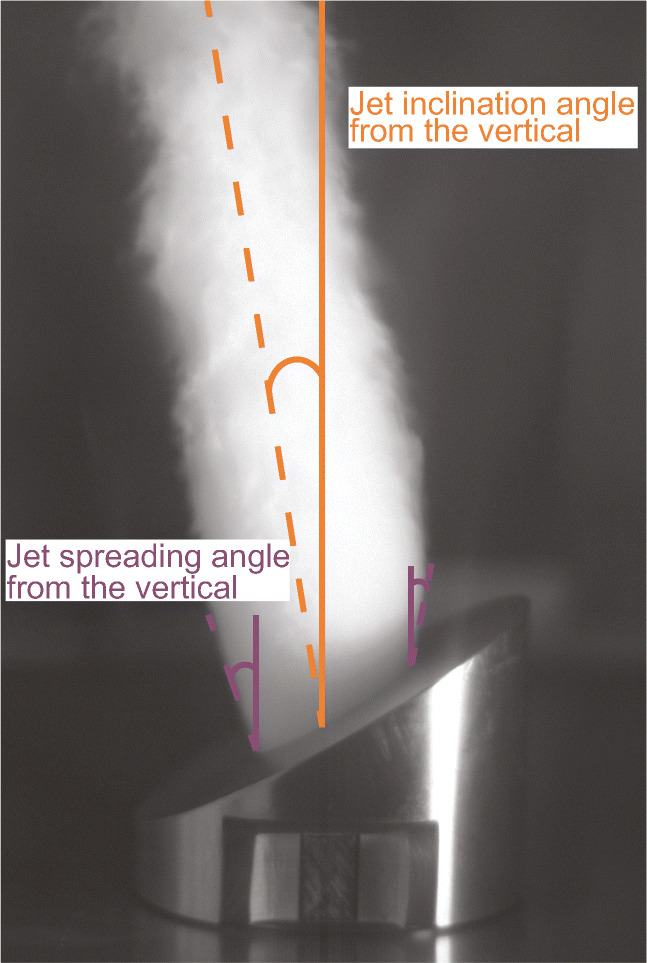
Measurement of gas spreading angle (purple) and jet inclination (orange). Gas spreading angle was always measured between vertical and a tangent at the jet boundary on the low and high vent exit. Jet inclination angle was measured as the deviation of the jet from the vertical centreline above the vent

Scaled single frames were exported and manually analysed with ImageJ. The gas spreading angle (Fig. 4, purple) was always measured between the vertical and a tangent at the jet boundary at the lower and higher vent side. Jet inclination was determined as the deviation from the vertical centreline of the gas flow (Fig. 4, orange). Driving medium, starting pressure and temperature are controlled precisely and the geometry is constant. Several studies (e.g. Alatorre-Ibargüengoitia et al. 2011; Cigala et al. 2017; Kueppers et al. 2006b) showed the reproducibility of repetitive experiments with heterogeneity of natural samples having the biggest impact. Here, no samples were part of the experiments. The opening of the diaphragms is sometimes imperfect. The state of the ruptured diaphragms after each experiment was controlled visually and only experiments with diaphragm opening that did not satisfy our criteria were repeated. In our experiments, the largest source of inaccuracy is the subjective error when manually and optically determining the centre streamline and the boundary layer of the jet to measure its spreading and inclination. Hence, measurements of jet spreading and inclination were repeated at least twice with the selected experiment being analysed by two individuals for an unbiased assessment.

### Scaling

Two experiments are similar, if they have the same non-dimensional parameters. Two different explosions at vastly different scales, e.g. in nature and in the laboratory, are equivalent, if all non-dimensional parameters match. In practice, full similarity in all parameters at the same time is not possible. To evaluate the differences between nature and experiments, it is crucial that the dynamics of the explosion are at least comparable. Since our vents are a modification of the vent geometries used by Cigala et al. (2017), we employed the same non-dimensional analysis of the flow conditions. We focused on the Reynolds number (Re) and the Mach number (*M*) for our vent geometries to describe the fluid flow dynamics. Re represents the ratio of inertial to viscous forces in a flow and is defined as:

$$ \operatorname{Re}=\frac{\rho UL}{\mu } $$

where *ρ* is the fluid density, *U* is the fully expanded flow velocity, *L* is a characteristic length, e.g. the vent radius (in our case) (Clarke 2013) or the jet diameter (Kieffer and Sturtevant 1984), and *μ* is the viscosity at the temperature of the fully expanded condition. The reference quantities in our experiments were calculated by using the one-dimensional isentropic theory (Oswatitsch 1952) by estimating gas density, viscosity and flow velocity for our experimental temperature and pressures (see Table 1 for gas properties of argon). The Re for our experiments was between 2.22 × 10^7^ (cylindrical, 5 MPa) at the vent exit and 9.09 × 10^8^ (diverging, 25 MPa) at fully expanded conditions. Re for volcanic eruptions is reported to be between 10^5^ and 10^8^ (Clarke 2013) or as high as 10^11^ (Kieffer and Sturtevant 1984). Furthermore, this way of scaling has proven to be viable for rapid decompression experiments (e.g. Cigala et al. 2017; Dellino et al. 2014; Dioguardi et al. 2013).

**Table 1:** The fluid properties of argon at 25 °C at the experimental pressures used in this study. Density, viscosity, speed of sound, *C*_P_ (specific heat capacity at constant pressure), *C*_V_ (specific heat capacity at constant volume) and *R* (gas constant) have been retrieved from Linstrom and Mallard (2000). The heat capacity ratio (*γ*) was calculated as $\gamma =\frac{C_P}{C_V}$

Fluid data argon @ 25 °C							
Pressure (MPa)	Density (kg/m^3^)	Viscosity (Pa s)	sound speed (m/s)	*C*p	*C*v	*γ*	*R*
0.1	16.22	2.27E−05	322.33	0.52	0.31	1.67	209.15
5	82.91	2.38E−05	327.19	0.58	0.32	1.82	262.81
8	134.37	2.47E−05	332.65	0.62	0.33	1.91	297.92
15	255.55	2.77E−05	351.96	0.71	0.34	2.12	376.57
25	416.71	3.32E−05	393.89	0.80	0.35	2.30	450.94

The flow Mach number was estimated by the following relationship (Saad 1985):

$$ \frac{A_2}{A^{\ast }}=\left({\left(\frac{2}{\gamma +1}\right)}^{\frac{\gamma +1}{2\left(\gamma -1\right)}}\right)\frac{1}{M}{\left[1+\left(\frac{\gamma -1}{2}{M}^2\right)\right]}^{\frac{\gamma +1}{2\left(\gamma -1\right)}} $$

where *A*_2_ is the area of the exit (28 mm diameter for the cylindrical vents and 43 mm for the diverging vents; see Fig. 3 for the 2D representation of the exit area) and A^*^ the critical area (26 mm diameter). A^*^ is defined as the narrowest cross-sectional area the gas flow has to pass during expansion and is located at the top of the sample chamber. The exit (*A*_2_) to critical (A^*^) area ratios were 1.16 and 2.73 for the cylindrical and diverging inner geometry, respectively. The heat capacity ratio γ was estimated for each experimental pressure resulting in Mach numbers between 1.54 and 3.82 for the cylindrical inner geometry at 5 MPa and the diverging inner geometry at 25 MPa (Table 2). All values for Re and *M* presented here represent maximum values that are, due to the dynamic nature of these type of experiments, only valid at the beginning of the experiment.

**Table 2:** Non-dimensional numbers calculated cylindrical and diverging inner geometries at the experimental pressures used in this study. Mach number (*M*) was calculated at the lower vent exit height. Reynolds number (Re) was calculated at the throat of the vent, the lower vent exit height and at fully expanded conditions. The characteristic length used to calculate these values is the vent exit diameter (28 mm for cylindrical vents and 43 mm for diverging vents)

Pressure	*M*	Re		
	Exit	Throat	Exit	Fully expanded
Cylindrical				
5	1.5	2.22E+07	2.85E+07	3.31E+07
8	1.6	3.58E+07	4.77E+07	6.78E+07
15	1.6	6.73E+07	9.69E+07	2.21E+08
25	1.6	1.06E+08	1.64E+08	5.92E+08
Diverging				
5	3.1	2.22E+07	4.98E+07	5.09E+07
8	3.2	3.58E+07	9.48E+07	1.04E+08
15	3.5	6.73E+07	2.55E+08	3.40E+08
25	3.8	1.06E+08	5.51E+08	9.09E+08

## Results

**Fig. 5:**
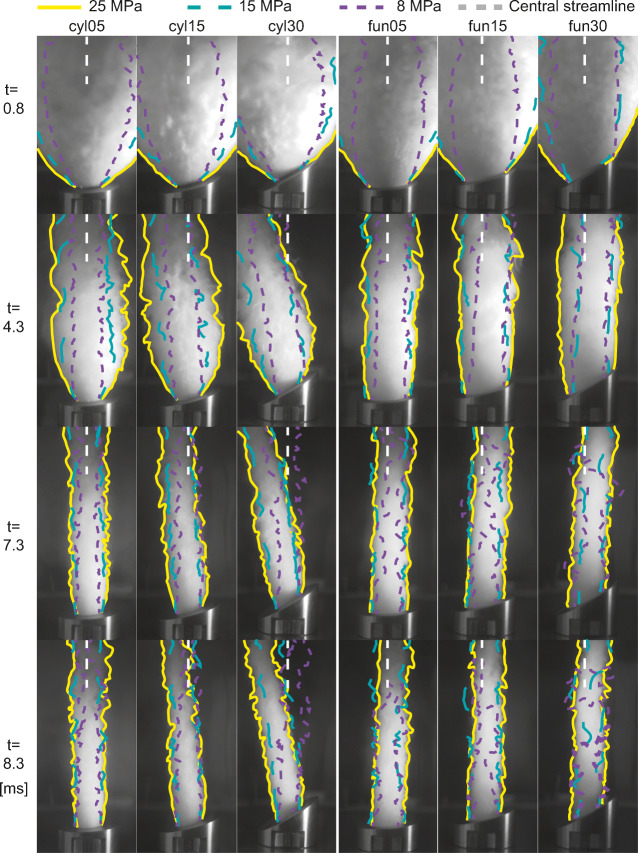
Gas-only experiments reveal a strong dependency of jet inclination to slant angle of the exit plane and reservoir pressure. Thereby, large slant angles and high-pressures cause a higher degree of tilt from the centreline. The six columns represent six different vent geometries (cyl05, cyl15, cyl30 have a cylindrical inner geometry and 5, 15 and 30° slant angle; fun05, fun15, fun30 are vents with 15° diverging inner geometry and 5, 15 and 30° slant angle respectively). The four rows represent different times after the first gas ejection (0.8, 4.3, 7.3, 8.3 ms). The coloured outlines mark different reservoir pressures (yellow 25 MPa, blue 15 MPa, purple 8 MPa). The underlying image shows an experiment at room temperature and 25 MPa initial overpressure

**Fig. 6:**
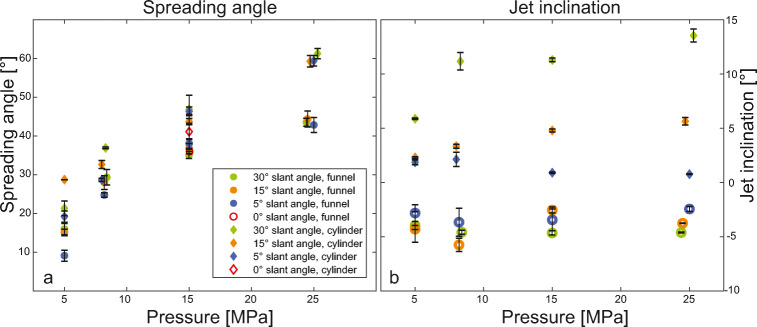
Gas jet spreading angle (6a) and jet inclination (6b) plotted against reservoir pressure. Initial pressure of 5, 8, 15 and 25 MPa. Circular symbols represent experiments with the 15° diverging vents and diamond symbols for cylindrical vents. The colours represent slant angle. Data for experiments with 0° slant angle taken from Cigala et al. (2017). **a** The spreading angle is highly affected by the initial reservoir pressure. Furthermore, higher slant angles of the exit plane produce bigger spreading angles. **b** The slant angle exerts the biggest control on jet inclination in experiments with the cylindrical geometry, while the initial reservoir pressure has no strong influence, except for the pressure increase between 5 and 8 MPa. For experiments with the diverging inner geometry, the jet inclination is around 5° against the dip direction of the exit. There seems to be no clear relationship between pressure ratio and/or slant and the degree of jet inclination

We focus here on the analysis of two features of the gas dynamics: *jet spreading* and *jet inclination*. Figure 5 illustrates the temporal evolution of jet dynamics as a function of autoclave overpressure and vent geometry. The colour-coded jet outlines represent three pressure starting conditions, columns and rows represent six geometries and four time intervals, respectively. We observed a strong influence of pressure ratio, slant angle and inner geometry on the dynamics of gas jets (Figs. 5 and 6). The images are still frames extracted from high-speed videos showing a condensing gas jet. In the first row, the images show the expanding gas 0.8 ms after the visual onset of gas ejection. The flow is choked at the system throat and underexpanded. At this time, asymmetry of gas expansion is visible via a larger extent of the jet towards the lower vent side (left side in Fig. 5). After 4.3 ms, gas jets from experiments starting at 25 and 15 MPa reservoir pressure are still underexpanded, while the initial overpressure in experiments with 5 and 8 MPa has already been accommodated. Now, jet asymmetry becomes even more apparent. The jets emitted from vents with the cylindrical inner geometry are inclined towards the side of the lower vent exit; jets emitted from the diverging vents are inclined to the opposite direction with an increasing inclination for diminishing underexpanded flow conditions.

By comparison of data from 7.3 and 8.3 ms, one can observe the effects of pressure decay of the reservoir. For jets produced by experiments with 8-MPa initial pressure, the boundary layer between jet and atmosphere becomes increasingly diffuse and the jet exhibits undulating motion at around 7.3 ms. In the case of the diverging geometry, the flow detaches from the vent when the gas spreading angle drops below the 15° slope angle of the diverging part of the geometry. After 8.3 ms, only the jets created from 25 MPa initial pressure are still underexpanded.

### Jet spreading

**Fig. 7:**
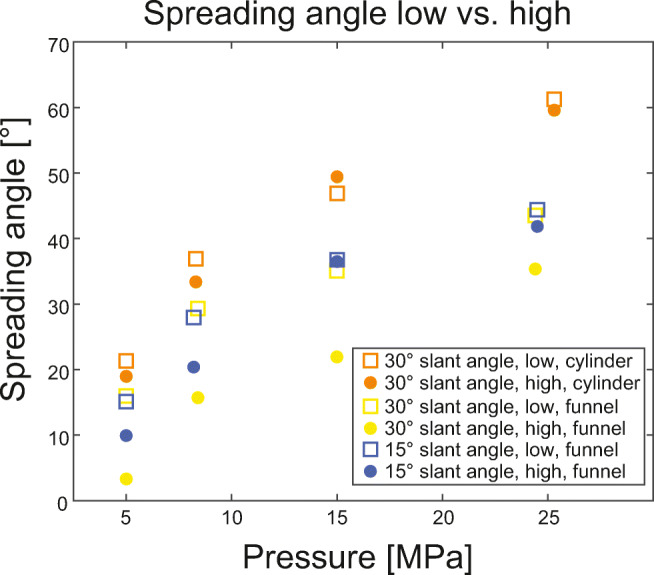
Maximum gas spreading angles on low and high vent exit side plotted against the reservoir pressure. Initial reservoir pressure of 5, 8, 15 and 25 MPa. Square symbols represent spreading angles on the lower vent exit side, circular symbols for spreading angles on the upper vent exit side. Orange symbols represent experiments with cylindrical geometry and 30° slant, yellow symbols diverging geometry with 30° slant angle and blue symbols for diverging geometry with 15° slant angle

The maximum gas jet spreading angle was sensitive to reservoir overpressure and slant angle of the exit plane (see Fig. 6a). Spreading angles evolved with time, showing a fast build-up to the maximum value and then a slower decay. Figure 6a reports the maximum spreading angle on the lower vent side that was achieved for individual experimental conditions. The pressure ratio was found to be of paramount influence on the maximum gas jet spreading angle, with higher pressure ratios causing larger spreading angles. Vents with cylindrical inner geometry had spreading angles that were, depending on reservoir pressure, between 5 and 20° larger than for diverging vents. Furthermore, for identical inner geometry and reservoir pressure, a positive correlation between spreading angle and slant angle as well as reservoir pressure was observed. When comparing results of setups 2 and 3, a positive correlation of initial gas reservoir volume and maximum spreading angle of the gas jet could be observed. The difference between maximum spreading angle on the lower and upper vent sides is controlled by inner geometry and slant angle (Fig. 7). For cylindrical vents, the slant angle has little effect on the spreading angle and the difference between lower and upper sides is generally small (around 2°). For diverging vents with 30° slant angle the difference is between 8 and 14° with the smallest difference in experiments with 25 MPa. For diverging vents with 15 and 5° slant angle the difference is generally smaller (between 0 and 8°, and 2–3°).

### Jet inclination

The emitted jet reacted to the slanted exit plane by deviating from the vertical centre streamline. We observed opposing effects of inner geometry on jet inclination direction: for cylindrical vents, jets were generally inclined in the dip direction of the vent surface (positive angles), whereas funnel vents showed the opposite trend (against the dip direction, negative angle, Fig. 6). For the case of vents with the cylindrical inner geometry, the strongest effect on jet inclination was exerted by the slant angle of the exit plane, followed by the pressure ratio. Overall, the maximum jet inclination was between 1° (for 5° slant and 5 MPa) and 13° (for 30° slant and 25 MPa). Jets emitted from vents with diverging geometry were generally less affected by slant angle or pressure ratio and their inclinations were between 2° and 7° for all cases. The smaller reservoir volume in setup 3 had no significant impact on jet inclination.

## Discussion

Volcanic explosions are the visible expression of a complex interplay of numerous source and path processes, several of which are likely to be highly variable with time. The crater may have an irregular shape. The vent may be open or clogged. Magma inside the conduit can exhibit strong textural gradients. The conduit may be vertical or inclined. Many of those parameters will be discussed in the following. Many studies have investigated the overall characteristics of eruption plumes. Here, we described the near-vent characteristics of gas jets in the gas-thrust region where the observed features are due to magmatic processes and air entrainment makes little or no contribution.

### Experiments

For each experiment, time zero is set at the visible onset of gas ejection from the main reservoir to account for subtle differences in diaphragm behaviour. The visibility of the gas is due to condensation upon expansion-driven cooling. After diaphragm rupture, the gas is expanding vertically and the flow requires some time to develop and generate quasi-static conditions for a short moment (Peña Fernández et al. 2020). As long as the jet is underexpanded at the vent (i.e. the gas pressure is above ambient pressure), the jet will expand horizontally. This study determined the maximum gas spreading angle as well as the jet inclination.

Two controlling factors with an influence on gas expansion dynamics were found: shallow subsurface geometry (inner geometry) and topography (slant angle, Fig. 3). The systematically larger spreading angle in experiments with cylindrical inner geometry is linked to higher pressure at the vent exit. Above the vent exit, gas can decompress radially. In experiments with diverging vents, radial gas expansion started inside the vent at the beginning of the diverging section (30 mm below the vent exit), resulting in systematically lower vent pressure. Similarly, the difference between the maximum spreading angle between lower and upper vent exit is also related to the vent exit pressure as the geometry affects the vertical difference between the two sides (Fig. 3). In the case of the cylindrical geometry, the maximum height difference between lower and upper vent exit is 16 mm (30° slant angle) but nearly twice as high (30 mm) for the diverging geometry. In summary, the dynamics in the gas-thrust region for volcanic eruptions, as well as our experiments, are strongly controlled by the ratio of exit area to critical area. Comparison with maximum gas spreading angles determined by Cigala (2017) for gas-particle jets shows similarity with gas spreading angles measured on the lower vent side (see Fig. 6a), but only for experiments where the onset of particle ejection (depending on particle to exit distance) starts after the maximum gas spreading angle has developed. Afterwards, the presence of particles at the vent exit alters flow conditions significantly. Adding particles to future experiments with slanted geometry will provide the opportunity for a thorough comparison of the effect of complex vent geometry on gas-particle ejection.

The jets from our vertical experiments were visibly inclined, showing a first-order influence of crater geometry on the gas-thrust region. In Fig. 6b, the jet inclination of experiments with cylindrical inner geometry and 5° slant angle appears to decrease with increasing pressure. However, the variation of inclination angles for the cyl05 is small and can be attributed to instabilities in the boundary layer that have a larger impact when the inclination angle is small. We observed the same two types of jet inclination behaviour as have been previously described numerically for sustained jets by Lagmay et al. (1999). The high spatial and temporal resolutions in our experiments with starting jets also reveal processes that have not been addressed before. Due to the dynamic nature of our experiments, we could observe the continuous transition caused by the depletion of the finite gas reservoir, involving variable degrees of jet underexpansion and supersonic flow conditions (Fig. 5). Overall, we observed jet inclination towards the lower vent side for cylindrical geometry and towards the upper vent side for diverging geometry, but already during underexpanded flow conditions. The two geometries show different inclination behaviour with time and starting overpressure. For cylindrical geometry, inclination was observed highest after 4.3 ms and was positively correlated with starting pressure and slant angle. For diverging geometry, the inclination increased with decreasing pressure but showed a clear asymmetry early on (e.g. Fig. 5, yellow outline, *t* = 4.3 ms). Flow instabilities towards the end of the visible gas flow overprint the jet inclination. In essence, vent geometry has been shown to cause asymmetry of the impulsive gas jets as it impacts gas expansion and air entrainment. Gas flow velocity and density changes due to entrainment affect plume buoyancy and ballistic pathways and should be included in the hazard assessment of primary volcanic risks.

### Volcanological implications

Volcanic eruptions are complex processes that have remained incompletely deciphered. Many boundary conditions cannot be measured directly and have to be measured remotely or estimated through model or experiments. Our scaled experiments revealed that the surface manifestation of a volcanic explosion can show directionality, even with a vertical and symmetrical subsurface geometry. In this simplified case, the direction of the jet is solely dependent on vent geometry and vent exit pressure. In nature, the dependencies are certainly more complex but assuming a vertical conduit and knowing the geometry of the vent, we might be able to make assumptions about the exit pressure based on observations of the emitted jets.

Asymmetrical gas-particle jets and eruption plumes have been described for large, pyroclastic density current issuing eruptions (Cole et al. 2015; Lagmay et al. 1999; Major et al. 2013). Inclined jets have also been observed for less energetic eruptions for example, at Stromboli Volcano, Italy, where inclination of the shallow plumbing system beneath the active craters in February 2004 was proposed by Zanon et al. (2009). Nine out of twenty observed jets exhibited a dip of around 7–13° towards the northwest regardless of wind direction. They stated that the inclined jets could be generated by a combination of deep-seated slug bursts within an inclined conduit. However, it was also reported that the morphology of the Northeast Crater was characterised by a deep and wide opening in the north-western crater wall at the time of the survey (Zanon et al. 2009). This kind of crater asymmetry is equivalent to those reported by Lagmay et al. (1999) and might deserve some consideration in accounting for the jet inclination for supersonic jets at Stromboli Volcano. In fact, the idea of an inclined shallow feeder system at Stromboli Volcano was previously proposed (Chouet et al. 2003) but the behaviour we observed in experiments with diverging geometry could account for vertical jets even with an inclined conduit. James et al. (2004) have described the influence of cylindrical conduit inclination on gas bubble ascent processes, leading to varying overpressure conditions at bubble burst and acentric rupture of the liquid film. When applied to higher viscosity magma, inclined conduits may ease a mechanical separation of gas bubbles and melt and enhance ascent velocity. There have been cases where an explosion destroyed parts of a symmetrical cone resulting in an immediate change from vertical jets to inclined jets (Schmid et al. 2020, in preparation). In such cases, it is unlikely that the conduit geometry changed over such a short timescale and hence, vent asymmetry must be the factor governing the directionality

The coupling of juvenile tephra to the (initially) surrounding gas jet is dependent on size and density (Taddeucci et al. 2017). Upon ejection into the atmosphere, the trajectory may be independent but the starting acceleration with a certain directionality is surely affected by vent geometry, making it a first-order parameter to consider for hazard assessment. An asymmetric vent with a variable exit height allows flatter trajectories and therefore a higher range of ballistics on sides with a lower vent exit height. The areas that can be affected by impacts of ballistics are thus skewed towards the lower side of the vent given a shallow explosion source. The difference in jet spreading angle on different sides of asymmetric volcanic vents could lead to a variance of entrainment efficiency. Hence, the likelihood of a column collapse towards the side with the smaller jet surface area (smaller spreading angle) might be elevated. The effect of inclined jets on pyroclast dispersal, as already observed (Cole et al. 2015; Lagmay et al. 1999; Major et al. 2013), adds another controlling force on the distribution of proximal hazards of explosive volcanic eruptions. Jet inclination seems to be exclusively governed by the pressure at the vent exit and the vent geometry. The latter (and its temporal variations) can be achieved today with a high resolution. Quantifying the vent exit pressure is less straightforward. It requires assumptions on pressure radiation in complex topography (Lacanna and Ripepe 2020) or near-exit measurements (Kueppers et al. 2019). Future measurements of gas dynamics in the near-vent gas-thrust region of volcanic explosions shall contribute to refined vent exit conditions. Some crucial parameters of volcanic vents affecting jet and plume behaviour can be constrained rapidly, reliably and with a high time resolution. Coupled with general knowledge from larger-scale observations of buoyant plumes and ballistic distribution, this will hopefully lead to enhanced hazard assessment as topographic variations may a priori allow to constrain size and location of areas of elevated risk.

## Concluding remarks

In summary, the morphology of volcanic vents and the overpressure affect the gas dynamics in the near-vent part of the gas-thrust region. Experiments with impulsive gas jets released from a vertical, cylindrical reservoir revealed the following positive correlations: The *pressure ratio* correlates positively with (1) the maximum spreading angle of the gas jet, (2) the maximum jet inclination for cylindrical vents and (3) the duration of underexpanded character of the jet. The *slant angle* correlates positively with (1) the maximum spreading angle of the gas jet and (2) the maximum jet inclination for cylindrical vents. Moreover, the *inner vent geometry* influenced the direction of the jet inclination in two distinct ways, (1) towards the direction of the exit plane dip for cylindrical vents and (2) against the direction of the exit plane dip for diverging vents. Additionally, cylindrical vents produced larger spreading angles then diverging vents. The *reservoir volume* showed positive correlation with maximum gas spreading angle but no significant impact on maximum jet inclination.

We demonstrate here that inner and outer vent and/or crater geometry can lead to inclined jets and asymmetrical jet spreading angles. Even though this is not commonly reported for volcanic eruptions, there are examples where crater asymmetry led to asymmetrical behaviour in the gas-thrust region and consequently in the areas affected by the eruption (Cole et al. 2015; Lagmay et al. 1999; Major et al. 2013).

Today, asymmetry of the vent and/or crater area can easily be detected and characterised by drone observations. Structure from motion photogrammetry allows the acquisition of data with unprecedented detail to analyse geometry, elevation, position and volumetric changes and their temporal evolution. Since this data collection is fast, easy, cheap and safe, even in times of volcanic unrest or ideally as part of a standard monitoring routine, asymmetry should not be neglected as factor influencing the proximal hazards of explosive volcanic eruptions.
